# Correlation between TyG index and its modified index with breast cancer risk in women a study based on NHANES database and real-world data

**DOI:** 10.3389/fonc.2026.1791080

**Published:** 2026-07-21

**Authors:** Qinbo Zhan, Huiliang Xu, Li Tang, Na Li, Da Miao

**Affiliations:** Department of Oncology, Shaoxing Second Hospital, Shaoxing, Zhejiang, China

**Keywords:** breast cancer, correlation, modified index, NHANES, TyG index

## Abstract

**Objective:**

To evaluate the association between the triglyceride-glucose (TyG) index and its modified indices with the risk of female breast cancer using data from the National Health and Nutrition Examination Survey (NHANES) database and to validate these findings with real-world data.

**Methods:**

Based on 1,086 women from the 2015 to 2016 NHANES cycle, the participants were divided into a breast cancer group (n = 32) and a non-breast cancer group (n = 1,054). The TyG index was calculated using the fasting glucose and triglyceride levels. TyG-BMI, TyG-WC, and TyG-WHtR were derived by incorporating the BMI, waist circumference, and height, respectively. Univariate and multivariate logistic regression analyses were used to assess the association between these indices and the risk of breast cancer. A nomogram was developed and its diagnostic performance was evaluated using calibration curves and ROC analysis. External validation was performed using real-world data through confusion matrix analysis.

**Results:**

Weighted analysis of the NHANES data revealed significantly higher median values of age, TyG, TyG-BMI, and TyG-WC in the breast cancer group than in the non-breast cancer group (P < 0.05). Inclusion of these variables in the multivariate logistic regression analysis identified age, TyG, TyG-BMI, and TyG-WC as factors associated with increased odds of breast cancer (P < 0.05). A nomogram model constructed using these factors indicated that advanced age, high TyG level, high TyG-BMI, and low TyG-WC were associated with increased odds of breast cancer (P < 0.05). Calibration curves for the training and testing sets of the nomogram prediction model demonstrated good agreement between the predicted and observed probabilities, with AUC values of 0.807 (95% confidence interval [CI]: 0.719–0.894) and 0.798 (95% CI: 0.702–0.894), respectively. External validation using real-world data showed that all metrics in the confusion matrix of the nomogram model exceeded 70%, indicating a good predictive performance.

**Conclusion:**

Advanced age, elevated TyG level, high TyG-BMI, and low TyG-WC were associated with increased odds of breast cancer. The clinical application of this model may facilitate the early identification of patients at a high risk of breast cancer.

## Introduction

Breast cancer is the most common malignant tumor among women. In 2020, approximately 2.26 million new cases were diagnosed globally, accounting for 11.7% of all cancer cases and 685,000 related deaths (1). New breast cancer cases in China account for 18% of the global total, and the number of new cases continues to increase (2). Research indicates that the age-standardized mortality rate for breast cancer attributable to metabolic risk factors in China is increasing, exceeding the global average (3). Developing practical and reliable non-invasive biomarkers to enable the early identification of high-risk individuals is of significant practical importance for advancing breast cancer diagnosis, reducing incidence and mortality rates, and alleviating the economic and psychological burdens on patients and their families.

In recent years, the widespread prevalence of unhealthy lifestyles has led to a rapid increase in the exposure to metabolic risk factors. These primarily include earlier age at menarche, later age at natural menopause, increased estrogen exposure, fewer pregnancies, and a shorter breastfeeding duration. Among these, breast cancer mortality associated with elevated body mass index (BMI) is particularly substantial (4, 5).

The triglyceride-glucose (TyG) index has emerged as a novel tool for identifying metabolic disorders (6). This method offers advantages, such as effectiveness, good reproducibility, ease of implementation, and low cost, making it readily applicable in clinical settings (7, 8). Studies have indicated that the TyG index can distinguish between benign and malignant breast lesions (9). Additionally, research has linked the TyG index to an increased risk of breast cancer, with findings showing that each one standard deviation increase in TyG is associated with a 1.36-fold higher risk of breast cancer (10). However, studies examining the association between modified TyG indices and breast-related conditions are scarce.

Therefore, this study aimed to evaluate the association between the TyG index and its modified indices with breast cancer risk in women, using data from the NHANES database. It further assessed their predictive performance for breast cancer, seeking to provide data references for early screening and precision intervention in breast cancer.

## Materials and methods

### Data selection and study design

**Figure 1:**
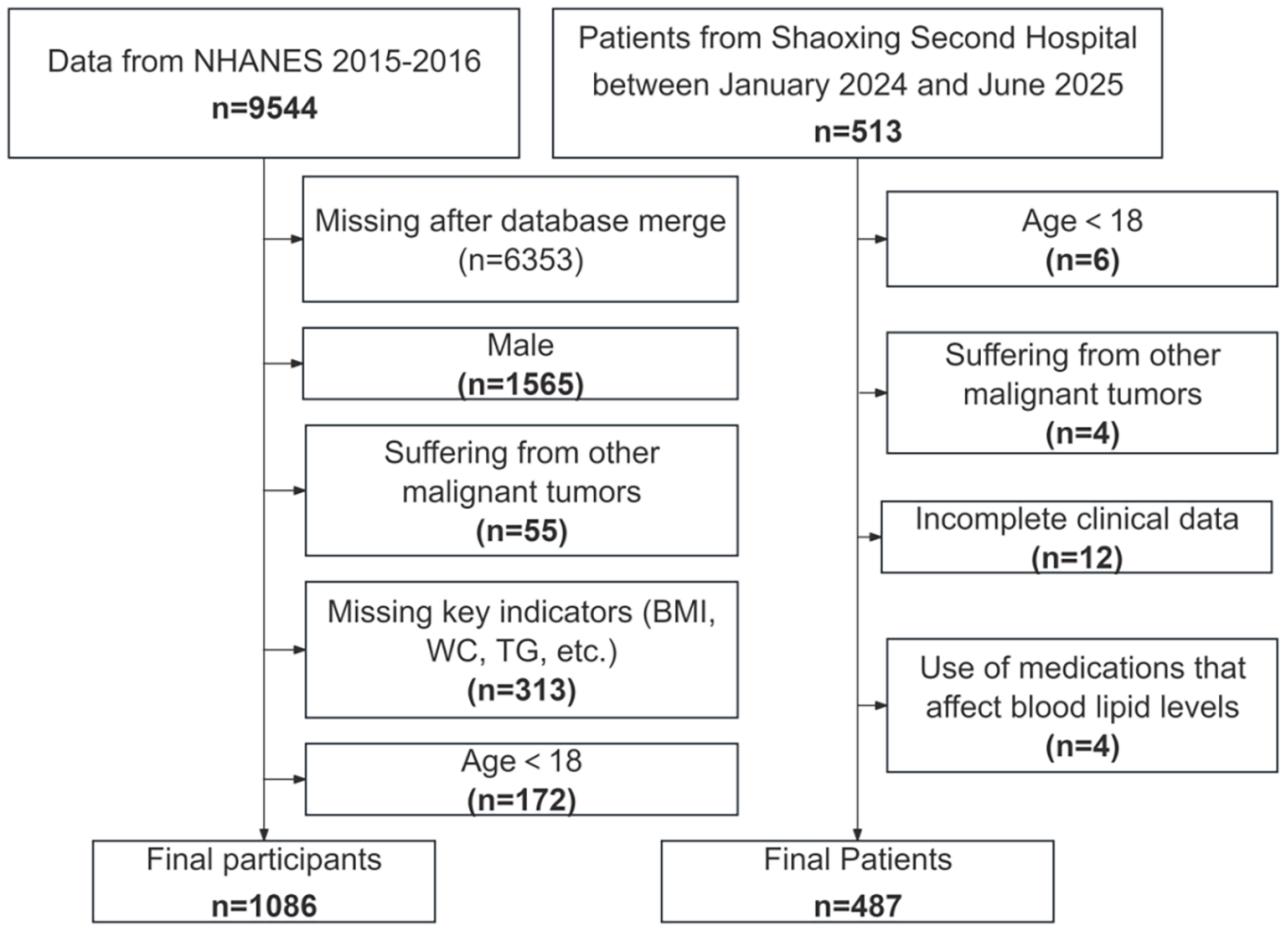
Study subject screening flowchart.

NHANES is a nationwide survey conducted by the National Center for Health Statistics (NCHS) using a stratified, multistage probability sampling design to comprehensively collect data on the nutritional status and health risk factors of the U.S. population. All participants provided informed consent, eliminating the need for additional ethical approval or consent. This study utilized data from the 2015–2016 cycle of the NHANES database, including 9,544 participants. The exclusion criteria were as follows: (1) male participants, (2) participants with missing key indicators (including fasting blood glucose, triglycerides, height, BMI, and waist circumference), (3) participants with other concurrent malignancies, and (4) participants aged <18 years. Ultimately, 1,086 eligible participants were included (**Figure 1**).

An additional 487 female patients treated at Shaoxing Second Hospital between January 2024, and June 2025 were enrolled, including 28 patients with confirmed breast cancer. Baseline data and clinical biochemical indicators were collected for all patients and the TyG index and its modified indices were calculated. The inclusion criteria were as follows: (1) age ≥ 18 years, (2) complete clinical records, and (3) informed consent obtained from both patients and their families. The exclusion criteria were as follows: (1) cognitive impairment, (2) concurrent malignant tumors, and (3) use of medications that affect lipid levels. This study was approved by the Ethics Committee of the Shaoxing Second Hospital.

### Group and weighting

Based on the 2015–2016 MCQ_I data from the NHANES database, participants were categorized into a breast cancer group (n = 32) and a non-breast cancer group (n = 1,054) according to the cancer status reported in MCQ220 (Ever told you had cancer or malignancy) and the cancer type reported in MCQ230 (What kind of cancer)?. Samples from this cycle were weighed using 2015–2016 examination weights (WTMEC2YR).

### Formula calculation

(1) TyG = ln[fasting TG (mg/dL) × fasting blood glucose (mg/dL)/2],

(2) TyG-BMI = TyG × BMI;

(3) TyG-WC = TyG × waist circumference (cm);

(4) TyG-WHtR = TyG × [waist circumference (cm)/height (cm]).

### Statistical analysis

Continuous variables with a normal distribution are expressed as x ± s, and intergroup comparisons were performed using the independent sample t-test. Continuous variables with skewed distributions are expressed as M (P25, P75), and intergroup comparisons were conducted using nonparametric tests. Categorical variables are presented as n (%), and intergroup comparisons were performed using the chi-squared (χ²) test.

Multivariate logistic regression analysis was employed to investigate the associations between the TyG index and its modified indices and the risk of breast cancer in women. The area under the receiver operating characteristic (ROC) curve (AUC) was used to evaluate the classification performance of the TyG index and its modified indices. Real-world data were collected for external validation using a confusion matrix. All statistical analyses were performed using R (version 4.3.0), with P < 0.05 considered statistically significant.

## Results

### Univariate analysis

Weighted analysis of the NHANES data revealed that the median values of age, TyG, TyG-BMI, and TyG-WC were significantly higher in the breast cancer group than in the non-breast cancer group (P < 0.05). No significant differences were observed in the remaining indicators (P > 0.05) (**Table 1**).

**Table 1:** Univariate analysis of weighted characteristics in the breast cancer study population.

Items	Non-Breast cancer group (n=1054)	Breast cancer group (n=32)	*Z/x^2^*	*P*
Age(years)	47.00(31.00,61.00)	65.00(57.25,76.00)	5.493	<0.001
BMI (kg/m2)	28.90(24.30,34.20)	28.85(24.00,34.63)	0.127	0.899
Smoke			0.064	0.800
Yes	536(50.85)	17(53.13)		
No	518(49.15)	15(46.87)		
Drink			1.862	0.172
Yes	563(53.42)	21(65.63)		
No	491(46.58)	11(34.37)		
Medical history			1.446	0.229
Yes	647(61.39)	23(71.88)		
No	407(38.61)	9(28.12)		
Race/Hispanic origin			–	0.267*
Mexican American	188(17.84)	6(18.75)		
Other Hispanic	154(14.61)	8(25.00)		
Non-Hispanic White	321(30.46)	11(34.38)		
Non-Hispanic Black	239(22.68)	3(9.38)		
Other Race	152(14.42)	4(12.50)		
Country of birth			0.789	0.374
Born in 50 US states or Washington, DC	705(66.89)	19(59.38)		
Others	349(33.11)	13(40.63)		
Marital status			–	0.182*
Married/Living with Partner	558(52.94)	19(59.38)		
Widowed/Divorced/Separated	262(24.86)	11(34.38)		
Never married	187(17.74)	2(6.25)		
Unknown	47(4.46)	0(0.00)		
Education level			–	0.546*
Less than 9th grade	169(16.03)	4(12.50)		
9-11th grade	100(9.49)	1(3.13)		
High school graduate/GED or equivalent	212(20.11)	6(18.75)		
Some college or AA degree	310(29.41)	9(28.13)		
College graduate or above	263(24.95)	12(37.50)		
Annual family income($)			–	0.197*
<25000	257(24.38)	7(21.88)		
25000~54999	383(36.34)	8(25.00)		
≥55000	358(33.97)	13(40.63)		
Unknown	56(5.31)	4(12.50)		
TC(mg/dL)	191.31 ± 40.91	193.75 ± 35.81	0.333	0.739
HDL-C(mg/dL)	59.79 ± 17.5	62.69 ± 17.29	0.922	0.357
LDL-C(mg/dL)	111.22 ± 35.23	109.84 ± 29.8	-0.219	0.826
TyG	8.38(7.92,8.86)	8.69(8.55,8.96)	3.758	<0.001
TyG-BMI	232.46(190.65,277.61)	254.13(209.17,308.19)	2.211	0.020
TyG-WC	805.36(682.37,924.45)	848.36(760.83,988.54)	2.093	0.048
TyG-WHtR	5.06(4.28,5.83)	5.21(4.74,6.33)	1.943	0.198

### Multivariate logistic analysis

Multivariate logistic regression analysis was conducted with breast cancer status (1 = yes, 0 = no) as the dependent variable, and age (original value), TyG (original value), TyG-BMI (original value), and TyG-WC (original value) as independent variables. The results indicated that age, TyG, TyG-BMI, and TyG-WC were factors associated with increased odds of breast cancer (P < 0.05). The regression model was logistic (P) = -12.007 + 0.068 × age + 0.743 × TyG + 0.021 × TyG-BMI − 0.008 × TyG-WC, as shown in **Table 2**.

**Table 2:** Multivariate logistic analysis.

Items	β	SE	Wald	P	OR	95%CI
Age	0.068	0.014	23.677	<0.001	1.071	1.042-1.101
TyG	0.743	0.374	3.940	0.047	2.103	1.009-4.380
TyG-BMI	0.021	0.008	6.581	0.010	1.021	1.005-1.038
TyG-WC	-0.008	0.004	5.574	0.018	0.992	0.985-0.999
Constant	-12.007	2.718	19.516			

### Nomogram model

**Figure 2:**
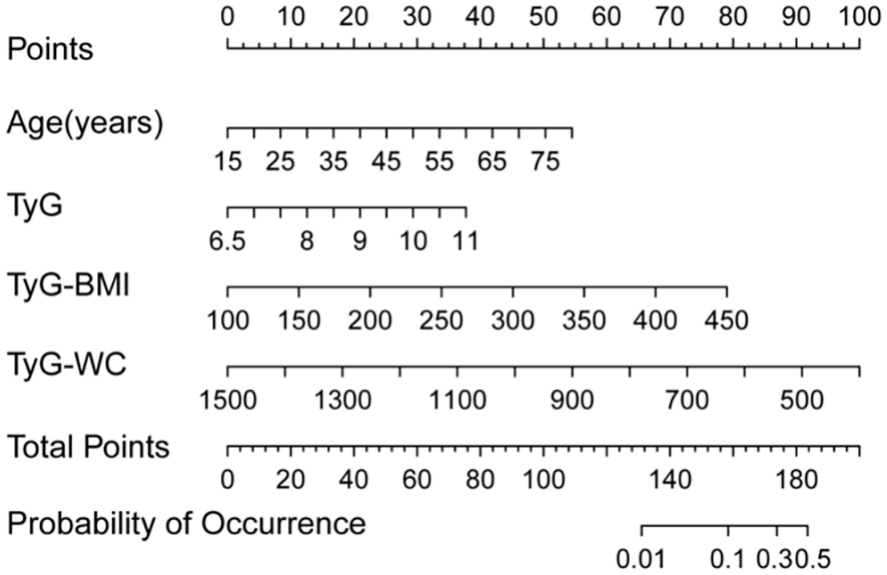
Nomogram model.

**Figure 3:**
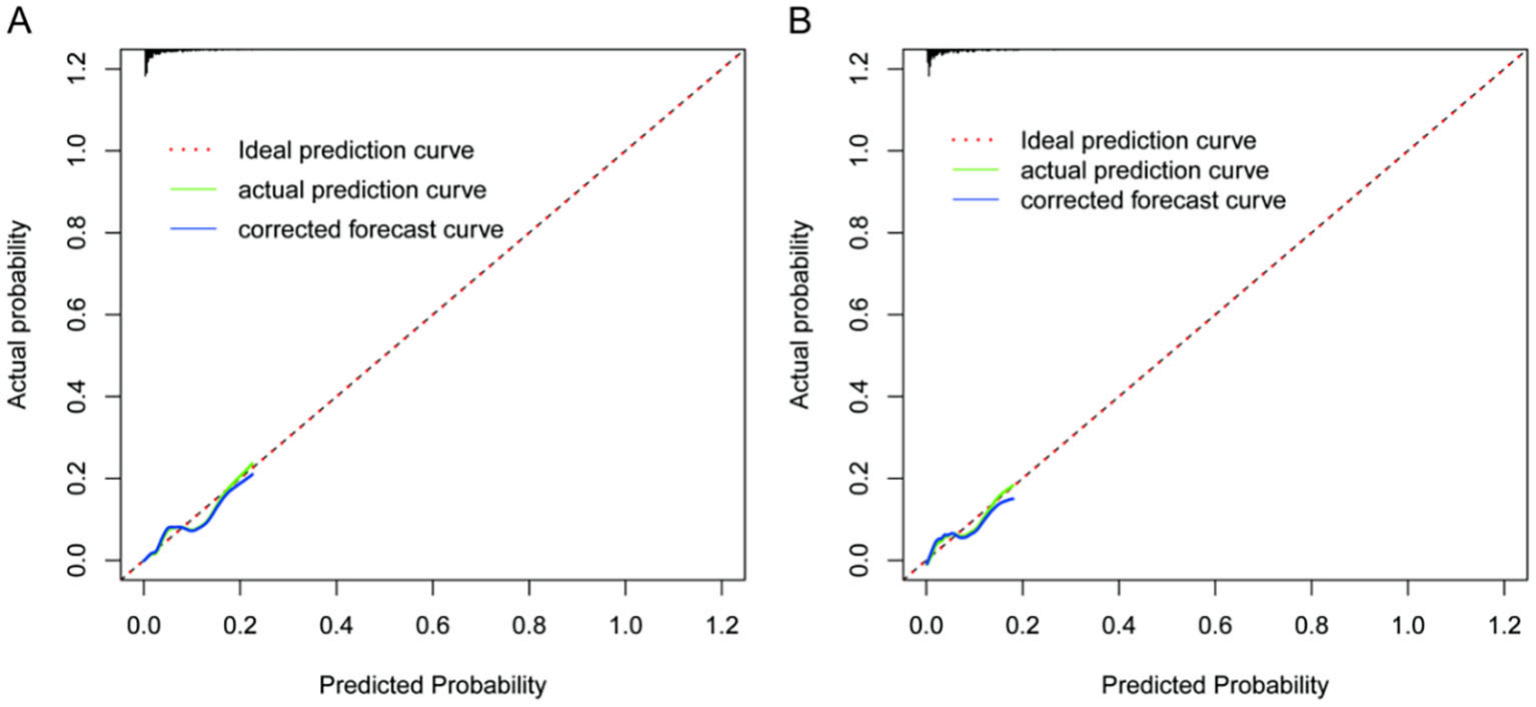
Calibration curve. **(A)** Train; **(B)** Test.

Variables with P < 0.05 in the multivariate logistic analysis were incorporated into the construction of the nomogram model. The results indicated that age, TyG, TyG-BMI, and TyG-WC were associated with increased odds of breast cancer (P < 0.05) (**Figure 2**). The calibration curves for the training and testing sets of the nomogram model demonstrated reasonable agreement between the predicted and observed probabilities, although the maximum range was relatively narrow (**Figures 3A, B**).

### ROC curve

**Figure 4:**
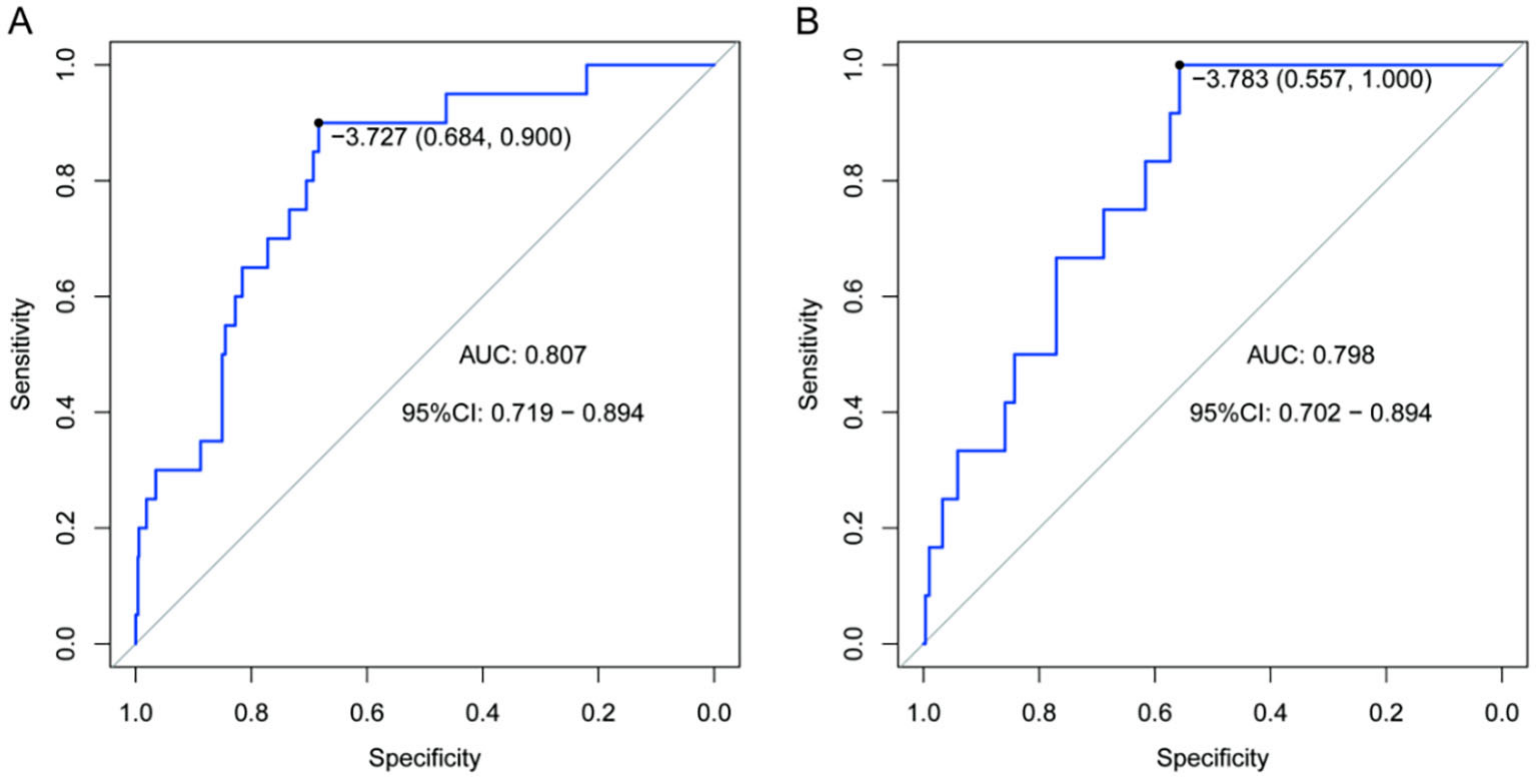
ROC curve. **(A)** Train; **(B)** Test.

The ROC curves for the breast cancer prediction model are shown in **Figures 4A, B**. The results indicated that the AUC values for the training and testing sets were 0.807 (95% CI: 0.719–0.894) and 0.798 (95% CI: 0.702–0.894), respectively.

### External validation confusion matrices

**Figure 5:**
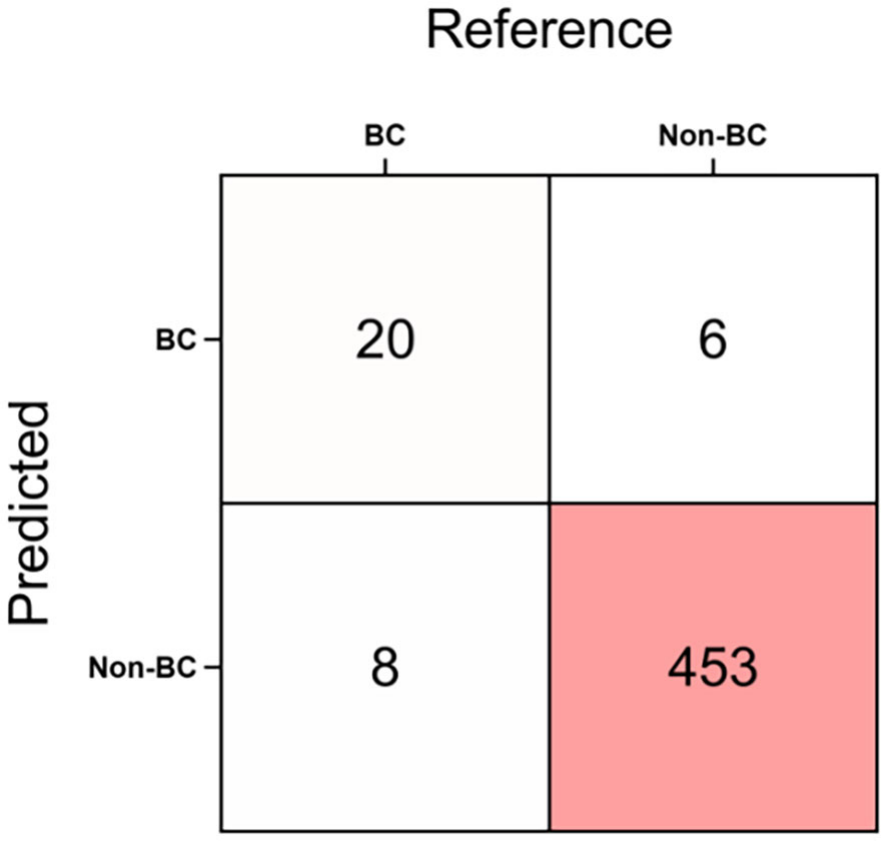
Confusion matrix.

Data from 487 patients at the Shaoxing Second Hospital between January 2024 and June 2025 were collected for external validation (**Figure 5**). The confusion matrix metrics of the nomogram model are listed in **Table 3**, indicating a good predictive performance.

**Table 3:** External validation metrics of confusion matrix(%).

Model	Accuracy	Sensitivity	Specificity	Precision	Recall	F1
Nomogram model	97.13	76.92	98.26	76.92	71.43	74.07

## Discussion

TyG serves as an alternative indicator for assessing insulin resistance (IR) based on fasting lipid and glucose levels. This metric reflects systemic insulin sensitivity by integrating the core parameters of lipid and glucose metabolism (11). Within the pathophysiological mechanisms of metabolic syndrome (MS), IR is regarded as the initial trigger and pivotal component in MS pathogenesis (12). MS manifests as a pathological state involving multiple metabolic dysregulations of proteins, lipids, and carbohydrates. Clinically, it is characterized by obesity, dyslipidemia, hyperglycemia, and hypertension. Moreover, MS and its associated biological markers significantly influence the development, treatment response, and prognosis of breast cancer (13, 14). Studies have identified IR as an independent factor affecting the occurrence and prognosis of malignancies of the female reproductive system (15).

To precisely evaluate the synergistic effects of obesity and metabolic abnormalities, researchers have combined the TyG index with obesity parameters to develop several derivative indices, including TyG-WC (waist circumference), TyG-BMI (body mass index), and TyG-WHtR (waist-to-height ratio) (16). Multiple studies have demonstrated that the TyG index and its modified indices are high-risk factors for breast cancer (17–19). Consequently, the TyG index and its modified indices are promising as important indicators for predicting breast cancer occurrence.

The results of this study indicated that age, TyG, TyG-BMI, and TyG-WC were significantly higher in the breast cancer group than in the non-breast cancer group (P < 0.05). After incorporating these variables into a multivariate logistic regression analysis, all variables were found to be significantly associated with breast cancer. The constructed nomogram model indicated that advanced age, high TyG level, high TyG-BMI, and low TyG-WC were factors associated with increased odds of breast cancer (P < 0.05). Elevated TyG and TyG BMI indices suggest potential insulin resistance and compensatory hyperinsulinemia (20). Insulin resistance indicates a possible chronic inflammatory state accompanied by fluctuations in sex hormone levels (21). Concurrently, elevated insulin levels may activate the insulin-like growth factor-1 (IGF-1) receptor pathway, promoting proliferation and inhibiting apoptosis in mammary epithelial cells. Consequently, high TyG and TyG BMI indices may be associated with the presence of breast cancer.

This discrepancy may arise because waist circumference reflects central obesity, and in patients with existing metabolic abnormalities, risks attributable to central obesity might be masked by TyG and TyG-BMI indices. However, this may be due to the larger sample size in the non-breast cancer group and the greater variation in TyG-WC within that group. The AUC values for the training and testing sets of the nomogram model constructed based on these variables were 0.807 and 0.798, respectively. In the external validation, all indicators exceeded 70%, indicating that the model had good predictive efficacy. When applied clinically, it can be used to identify high-risk patients who may develop breast cancer at an early stage using readily accessible metabolic indicators.

In summary, advanced age, high TyG level, high TyG-BMI, and low TyG-WC are factors associated with increased odds of breast cancer. Their clinical application provides readily accessible screening indicators for early identification of high-risk patients.

## Conclusion

Advanced age, high TyG level, high TyG-BMI, and low TyG-WC are factors associated with increased odds of breast cancer. Clinically, these readily accessible screening indicators can facilitate early identification of high-risk patients. This study conducted a cross-sectional analysis using the NHANES database. However, since data on breast cancer in the NHANES database are limited and have not been updated since 2016, data from 2017 onward could not be analyzed. In addition, due to the small number of positive events in the database, the EPV is slightly below the ideal threshold, furthermore, the database lacks pathological information on breast cancer, leaving potential confounding factors unaccounted for (e.g., differences in indicators across various stages and subtypes of breast cancer). Consequently, in-depth subgroup analyses were not feasible. Additionally, the unclear timing of indicator measurements within the NHANES database may have introduced some degree of bias into the final results. Future multicenter studies should collect more detailed and in-depth data.

## Data Availability

The original contributions presented in the study are included in the article/supplementary material. Further inquiries can be directed to the corresponding author.
